# Expression profile and specific network features of the apoptotic machinery explain relapse of acute myeloid leukemia after chemotherapy

**DOI:** 10.1186/1471-2407-10-377

**Published:** 2010-07-19

**Authors:** Marco Ragusa, Giuseppe Avola, Rosario Angelica, Davide Barbagallo, Maria Rosa Guglielmino, Laura R Duro, Alessandra Majorana, Luisa Statello, Loredana Salito, Carla Consoli, Maria Grazia Camuglia, Cinzia Di Pietro, Giuseppe Milone, Michele Purrello

**Affiliations:** 1 Dipartimento di Scienze BioMediche, Unità di BioMedicina Molecolare Genomica e dei Sistemi Complessi, Genetica, Biologia Computazionale G Sichel, Via Santa Sofia 87, 95123 Catania, Italy, EU; 2 Dipartimento di Scienze BioMediche, Sezione di Ematologia, 95125 Catania, Italy, EU

## Abstract

**Background:**

According to the different sensitivity of their bone marrow CD34+ cells to *in vitro *treatment with Etoposide or Mafosfamide, Acute Myeloid Leukaemia (AML) patients in apparent complete remission (CR) after chemotherapy induction may be classified into three groups: (i) normally responsive; (ii) chemoresistant; (iii) highly chemosensitive. This inversely correlates with *in vivo *CD34+ mobilization and, interestingly, also with the prognosis of the disease: patients showing a good mobilizing activity are resistant to chemotherapy and subject to significantly higher rates of Minimal Residual Disease (MRD) and relapse than the others. Based on its known role in patients' response to chemotherapy, we hypothesized an involvement of the Apoptotic Machinery (AM) in these phenotypic features.

**Methods:**

To investigate the molecular bases of the differential chemosensitivity of bone marrow hematopoietic stem cells (HSC) in CR AML patients, and the relationship between chemosensitivity, mobilizing activity and relapse rates, we analyzed their AM expression profile by performing Real Time RT-PCR of 84 AM genes in CD34+ pools from the two extreme classes of patients (i.e., chemoresistant and highly chemosensitive), and compared them with normal controls.

**Results:**

The AM expression profiles of patients highlighted features that could satisfactorily explain their *in vitro *chemoresponsive phenotype: specifically, in chemoresistant patients we detected up regulation of antiapoptotic BIRC genes and down regulation of proapoptotic APAF1, FAS, FASL, TNFRSF25. Interestingly, our analysis of the AM network showed that the dysregulated genes in these patients are characterized by high network centrality (i.e., high values of betweenness, closeness, radiality, stress) and high involvement in drug response.

**Conclusions:**

AM genes represent critical nodes for the proper execution of cell death following pharmacological induction in patients. We propose that their dysregulation (either due to inborn or *de novo *genomic mutations selected by treatment) could cause a relapse in apparent CR AML patients. Based on this, AM profiling before chemotherapy and transplantation could identify patients with a predisposing genotype to MRD and relapse: accordingly, they should undergo a different, specifically tailored, therapeutic regimen and should be carefully checked during the post-treatment period.

## Background

AML is heterogeneous both at the cytogenetic and the molecular level [1,2]. Many of these alterations have prognostic impact on the clinical outcome, especially on resistance to chemotherapy and relapse rates [1,2]. As with other tumours, one of the main obstacles to successful chemotherapy is drug resistance [3,4]. Nearly 80% of AML patients apparently achieve CR following chemotherapy induction, however a high proportion of them relapses and eventually dies of the disease [5,6]. To explain MRD or relapses in these patients after chemotherapy, it has been suggested that the bone marrow microenvironment may protect cancer hematopoietic cells, allowing them to resist treatment and survive [7]. This form of resistance (called *de novo *drug resistance) could be in part attributable to the interaction of the Very Late Antigen (VLA)-4 of leukemic cells with fibronectin within bone marrow stroma [8]. VLA-4 and other adhesion molecules are involved in homing and mobilization of hematopoietic stem cells (HSC) [9]. Treatment strategies, based on blocking adhesion molecules or other proteins involved in homing, might minimize MRD [10]. It has been demonstrated that patients that are *good mobilizers *are significantly more likely to be responsive to chemotherapy than *poor mobilizers *[10]. However, unlike other leukemic patients, *good mobilizers *CR AML patients are subject to significantly higher rates of relapse than patients with lower mobilization capacity; they also have a higher rate of MRD at remission [11-13]. A high ability to mobilize CD34+ cells in peripheral blood (PB) was proved to be an unfavourable prognostic factor, independent of others such as class of cytogenetic risk or FAB morphotype [11-13]. It has been hypothesized that intrinsic or acquired chemoresistance of leukemic bone marrow precursors may be a possible explanation for the association between high mobilization of CD34+ cells and MRD. Furthermore, it has been demonstrated that non-leukemic HSC from AML patients show differential chemosensitivity: this allowed the identification of three categories of patients, characterized by: (i) normal chemosensitivity, (ii) chemoresistance, (iii) increased chemosensitivity, respectively (Milone et al: Chemo-sensitivity of clonigenic marrow precursors in AML patients in complete remission: association with CD34+ mobilization and with disease-free survival, submitted). A statistically significant correlation between *in vitro *chemosensitivity of CFU-GM to Etoposide and Mafosfamide, CD34+ cells mobilization and survival was detected: patients with high mobilization capacity showed chemoresistance of their BFU-E and CFU-GM and lower survival rates than patients with normal or low mobilization activity. Dysregulation of the Apoptotic Machinery (AM) plays a primary role in the response to antineoplastic therapy [14,15]. In a previous work by some of us, it was demonstrated that the highly interconnected nodes of the AM network (hubs) typically represent the genes with the highest number of genome, transcriptome and proteome alterations in several cancer models [16]. Moreover, we found that most of AM proteins targeted by drugs were characterized by high connectivity; in particular, there was a highly significant association between the betweenness of these proteins and their being targets of drugs [16]. Based on these findings and on our data on AM involvement in anticancer treatment (i.e., Fenretinide in neuroblastoma and Imatinib in CML) [16], we explored the hypothesis that specific AM expression profiles in CR AML patients may determine the differential chemosensitivity, shown *in vitro *by their bone marrow HSC. The results allow us to propose that there is a relationship between the AM network specific structure and its proneness to dysregulation.

## Methods

The transcriptome of 84 human genes, assigned to the Apoptotic Machinery [17], was analyzed in bone marrow CD34+ cells from a cohort of AML patients in apparent CR and from normal controls: in all of them, BFU-E, CFU-E, CFU-GM hematopoietic clonogenic precursors were checked for *in vitro *chemoresistance. Chemosensitivity tests on freshly collected cells and molecular analysis after cell cryopreservation were performed on the same bone marrow samples. AML patients were selected from a cohort of 37 patients studied prospectively, in which the HSC *in vitro *chemosensitivity was determined. Based on their *in vitro *drug sensitivity in comparison to normal controls, patients were divided into three groups: (i) normally chemoresponsive, (ii) chemoresistant, (iii) highly chemosensitive. Three patients from the second group and three from the last one were selected for further detailed molecular analysis.

### Clinical Features of AML Patients

Patients' clinical characteristics are reported in Additional File 1. At the time of the study, all patients were in first apparent CR after a cycle of chemotherapy induction and consolidation. Remission was determined by cytogenetic analysis, cytofluorometry, immunophenotyping. The study was approved by the Institutional Ethics Committee and all patients and control donors gave their informed consent for participation in this research.

### CD34+ Cells Mobilization in PB of AML Patients

All patients were treated using a similar chemotherapeutic regimen: one or two induction cycles with antracycline, cytarabine (ARA-C) and etoposide, followed by a consolidation cycle with ARA-C and Mitoxantrone in CR patients. A group of normal bone marrow donors (n = 15) was also studied as control. CD34+ mobilization was performed in all CR AML patients after the induction phase. CR was confirmed at that time by immunophenotyping and by cytogenetic assessment in patients showing cytogenetic abnormalities at diagnosis. CD34+ mobilization was carried out during the recovery phase after the first consolidation cycle: G-CSF was administered at the dose of 10 mcg/kg/day sc, starting 10 days after the end of the consolidation cycle and until the end of the aphaeretic harvest. During mobilization, PB CD34+ cells were daily assessed from the day in which the WBC count in the PB was > 1 × 10^9^/L; the peak value of the CD34+ cells was used as end point for evaluating the mobilizing ability.

### Selection of CD34+ Cells

Mononuclear cells from bone marrow were purified on a Ficoll gradient at the time of *in vitro *chemosensitivity assays, frozen in FBS with 10% DMSO, and stored in liquid nitrogen. After thawing and washing with IMDM (containing 2% FBS) to remove DMSO, cells were incubated with DNAse to degrade free DNA in solution that would compromise separation efficiency. Separation of CD34+ cells was performed using the CD34 MicroBead Kit (Miltenyi Biotec Macs mini). A final cell purity higher than 80% was achieved.

### *In Vitro *ChemoSensitivity Assay Of Hematopoietic Precursors

Bone marrow aspiration in AML patients was carried out between the fourth and sixth week after the end of CD34+ cells mobilization, during disease evaluation. Bone marrow from normal donors was collected through the harvest procedure for allogeneic transplantation. Mononuclear cells were collected from bone marrow samples by Ficoll density gradient separation and divided into six aliquots of 10 million cells in TC199. After a short incubation at 37°C, cell aliquots were treated with increasing concentrations of Etoposide (40 and 60 mcg/ml) or Mafosfamide (50, 75, 100, 150 mcg/ml), whereas one aliquot was left untreated as control. Cells were incubated at 37°C for 30', washed in cold TC199 (4°C), resuspended in IMDM with 2% FBS, seeded (2 × 10^4^/ml) in methylcellulose medium supplemented with erythropoietin (HSC-CFU lite with EPO MEDIA MACS Miltenyi Biotec), and incubated in a 37°C, 5% CO2, humidified incubator. After 14 d, the colonies (BFU-E, CFU-E, CFU-GM) were analyzed by inverted light microscopy. Drug sensitivity was measured by comparing the number of hematopoietic colonies in samples treated with drugs to untreated controls.

### Real Time PCR

Due to the very low amount of cells at the end of the purification procedure, we had to pool CD34+ cells from three patients with an identical chemophenotype to perform our analysis. Total RNA was extracted by using FastPure™ RNA Kit (Takara), according to the Manufacturer instructions. 1 μg of total RNA was reverse-transcribed using the High Capacity RNA-to-cDNA Kit (Applied Biosystems). 10 ng of cDNA were added to each well of a 96 well PCR array for quantitative PCR (The Human Apoptosis RT^2 ^Profiler™ PCR Array, SuperArray Bioscience Corporation, MD, USA). The array consisted of 96 primers for 84 protein-encoding AM genes, plus five control genes (ACTB, B2 M, GAPDH, HPRT1, RPL13A), together with PCR and sample quality controls. PCR cycles were performed according to the manufacturer instructions. All experiments were performed in duplicate. Quantitative real time PCR was performed on a Mx3005P™ QPCR system (Stratagene, La Jolla, CA, USA). The threshold cycle (Ct), defined as the cycle number at which the amount of amplified target reaches a fixed threshold, was obtained for each gene in each sample. The Ct for each gene in each sample was normalized to the Ct of control genes, provided in the array, and with respect to each other (normal vs resistant; normal vs sensitive; resistant vs sensitive), according to the 2^-ΔΔCT ^method [17]. We also reported our data and the corresponding fold changes applying the following procedure (see Results): if the 2^-ΔΔCT ^(RQ) was ≥1, we used the same RQ number (the fold change is positive = up regulation); on the other hand, if RQ was < 1, we calculated -1/RQ. In this last case, the fold change was negative (down regulation). Analysis and visualization of data was obtained by using the MultiExperiment Viewer 4.4 [18]. To identify differentially expressed genes for the three different comparison reported above, we applied a t-test between subjects (ΔCt) by using the following parameters: assumption of equal variance; Alpha (overall threshold p-value) = 0.05; the p-value was based on t-distribution; the significance was determined by the Adjusted Bonferroni Correction. We reported as up- or down regulated genes having an expression fold change at least ≥ 2 and ≤ -2, respectively. Data were partitioned through Hierarchical Clustering, by using the Euclidean distance metric, and the Average Linkage Clustering as linkage method.

### Network Analysis

The global AM network was generated by retrieving, through cytoscape plug-in APID2NET [19,20], all the available experimental interactions among all the 84 AM genes analyzed through the Human Apoptosis RT^2 ^Profiler™ PCR Array. To create the three specific AM networks, based on three different patient class expression profiles, we applied on APID2NET-interaction maps the three different expression datasets from the chemoresponse classes, using both colour and size gradients of nodes to indicate expression fold changes. Analysis of network centrality was performed by using the plug-in Network Analyzer, which allows to retrieve all the centrality parameters of a node from an established network [21]. The degree of a node inside any biological network mirrors the general topological features of the network, not the real functional importance of the specific node. For this reason, we focused our analysis also on other centrality metrics (i.e., betweenness, closeness, radiality, stress): in biological terms, these may be interpreted as the probability of a protein to be functionally relevant for other proteins and its functional ability to connect different cell nodes. Mathematical details of these centrality metrics are reported by Brandes and Erlebach [22]. Data on functional interactions between genes and drugs were extracted from the Comparative Toxicogenomics Database (CTD), a manually curated repository of specific chemical-gene and chemical-protein interactions in vertebrates and invertebrates from published literature [23]. We inferred the possible correlation between dysregulation of AM genes and their network centrality (NC) or overall drug response (ODR, estimated through the number of literature citations for drugs) by comparing the different NC and ODR in the two different gene expression classes (differentially expressed genes and unchanged genes) by the Wilcoxon Rank Sum test (p-value< 0.05).

## Results

### *In Vitro *ChemoSensitivity Of Hematopoietic Precursors

In normal subjects, after incubation with maphosphamide at 50 mcg/mL, the mean percentage of normalized residual growth of CFU-GM was 45% (range 24% to 57%). In AML patients, the sensitivity of CFU-GM was highly heterogeneous: by using the values found in normal controls as cut-off points, three groups of patients could be identified. A first group was made of 13/37 (35%) AML patients, with a sensitivity in the range of normal controls and a mean residual CFU-GM of 33.8% (normally chemoresponsive patients). In 6/37 patients (16%), CFU-GM showed increased resistance with a residual CFU-GM above the upper limit of the normal range and a mean of 73.8% (chemoresistant patients). The third group comprised 18/37 (48%) AML patients with an increased sensitivity to maphosphamide, a residual growth of CFU-GM below the lower limit of the normal range and a mean of 6.2% (highly chemosensitive patients).

### AM Expression Profile in CD34+ Cells from CR AML Patients

**Figure 1:**
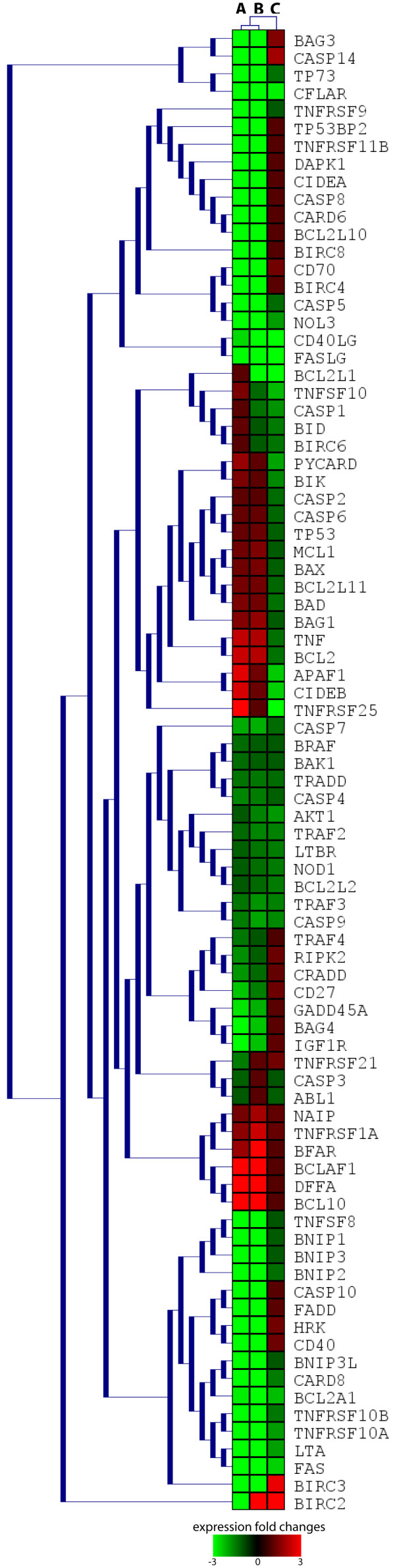
**Expression matrix of AM genes in CD34+ cells from CR AML patients**. Up regulated genes are in red, down regulated genes in green, according to the bar shown below the matrix. Each row represents the colour coded expression of a specific gene; the columns represent the colour coded AM profiles, obtained for each type of comparison between classes: A = chemosensitive patients vs controls; B = chemoresistant patients vs controls; C = chemoresistant vs chemosensitive patients. Hierarchical clustering of expression values is based on euclidean distances and average linkage.

Quantitative PCR array technology was exploited to examine the transcript levels of 84 AM genes in CD34+ pools from CR AML patients, exhibiting a chemoresistant or a chemosensitive phenotype after *in vitro *treatment, and control donors. Transcript quantification by the 2^-ΔΔCT ^method showed that 23 AM genes from the Bcl2, Birc, Bnip, caspases, death receptors, death ligands gene families had a nearly similar expression profile in both classes (Tables 1 and 2; Figure 1). On the other hand, 42 AM genes had a different expression profile in patients from either the chemoresistant or the chemosensitive class with respect to controls (Table 1; Figure 1). With the exception of BCL10, BCLAF1, DFFA, that were up regulated, all of these AM genes were down expressed with respect to controls (Table 1; Figure 1). Up regulation of proapoptotic genes BCL10, BCLAF1, DFFA and down regulation of antiapoptotic BAG3, BIRC4, BIRC8, BNIP1, BNIP2, CARD6, CD70, CFLAR, NOL3 in CD34+ bone marrow cells, from both chemoresistant and chemosensitive CR patients, strongly suggest that their AM molecular profile is prone to activation (Tables 1 and 2; Figure 1). We also found that proapoptotic DAPK1, TNFRSF11B, TNFSF8, TP73 genes were down regulated in CD34+ cells from our cohort of CR AML patients (Tables 1 and 2; Figure 1), as already reported for other leukaemia patients after chemotherapy [24-26]; we also observed a down regulation of antiapoptotic BAG3, CD70, NOL3 (Tables 1 and 2; Figure 1), that had been previously reported in patients with primary B chronic lymphocytic leukaemia after chemotherapy [27-29]. The comparison of the AM expression profile between resistant and sensitive samples also demonstrated the up regulation of BIRC2 and BIRC3 (two members of the antiapoptotic BIRC family) and down regulation of APAF1, BCL2A1, BCL2L1, CD40LG, CIDEB, FAS, FASL, TNFRSF25, TNFSF10 in the former class (Tables 1 and 3; Figure 1). This expression profile would appropriately explain the *in vitro *acquired chemoresistant phenotype of CD34+ cells from *high mobilizers*: in fact, up regulation of BIRC genes was already reported to be related to drug resistance in leukemias and other cancers, as well as the down regulation of proapoptotic APAF1, FAS, FASL, TNFRSF25 [30-34]. Contrary to data previously reported on *in vivo *resistant AML cells, we detected a down regulation of antiapoptotic BCL2A1 and BCL2L1, respectively, in *in vitro *chemoresistant CD34+ cells from our cohort of CR patients (Tables 1 and 3; Figure 1).

**Table 1:** Expression fold changes of 84 AM genes in CD34+ cells from CR AML patients

Genes	chemoresistant vs control	chemosensitive vs control	chemoresistant vs chemosensitive	Genes	chemoresistant vs control	chemosensitive vs control	chemoresistant vs chemosensitive
**ABL1**	1.05 (0.85)	-1.04 (1)	-1.11 (1)	**CASP7**	-2.08 (0.14)	-2 (0.03)	-1.26 (0.17)

**AKT1**	-1.55 (0.15)	-1.05 (0.87)	-1.79 (0.18)	**CASP8**	-15.74 (0.006)	-19.29 (0.04)	1.01 (1)

**APAF1**	1.35 (0.29)	2.66 (0.08)	-2.38 (0.007)	**CASP9**	-1.95 (0.20)	-1.46 (0.08)	-1.61 (0.09)

**BAD**	1.35 (0.26)	1.47 (0.20)	-1.32 (0.46)	**CD27**	-1.49 (0.24)	-2.33 (0.11)	1.29 (0.99)

**BAG1**	1.75 (0.34)	1.55 (0.47)	-1.07 (1)	**CD40**	-3.11 (0.02)	-4.89 (0.01)	1.3 (0.50)

**BAG3**	-41.26 (0.0004)	-76.11 (0.00009)	1.53 (1)	**CD40LG**	-16.3 (0.01)	-2.57 (0.04)	-7.67 (0.02)

**BAG4**	-2.34 (0.09)	-2.99 (0.08)	1.06 (1)	**CD70**	-10.31 (0.002)	-17.03 (0.01)	1.37 (0.62)

**BAK1**	-1.11 (1)	-1.26 (0.47)	-1.06 (1)	**CFLAR**	-51.86 (0.002)	-22.01 (0.003)	-2.85 (0.03)

**BAX**	1.43 (0.80)	1.31 (0.73)	-1.11 (1)	**CIDEA**	-15.74 (0.008)	-19.29 (0.009)	1.01 (1)

**BCL10**	3.09 (0.007)	3.01 (0.007)	1.01 (1)	**CIDEB**	1.26 (0.37)	2.55 (0.03)	-2.45 (0.001)

**BCL2**	2.15 (0.12)	1.9 (0.23)	-1.35 (0.30)	**CRADD**	-1.24 (0.18)	-1.77 (0.07)	1.18 (0.52)

**BCL2A1**	-5.19 (0.01)	-2.81 (0.02)	-2.23 (0.004)	**DAPK1**	-15.74 (0.0006)	-19.29 (0.0002)	1.01 (1)

**BCL2L1**	-3.5 (0.007)	1.06 (0.44)	-4.47 (0.005)	**DFFA**	3.11 (0.00008)	3.01 (0.00009)	1.01 (1)

**BCL2L10**	-15.74 (0.01)	-19.29 (0.02)	1.01 (1)	**FADD**	-5.02 (0.004)	-6.41 (0.004)	1.06 (1)

**BCL2L11**	1.36 (0.25)	1.38 (0.26)	-1.22 (0.72)	**FAS**	-7.19 (0.01)	-3.53 (0.04)	-2.46 (0.003)

**BCL2L2**	-1.25 (0.24)	-1.04(1)	-1.44 (0.21)	**FASLG**	-22.58 (0.005)	-4.32 (0.02)	-6.32 (0.01)

**BCLAF1**	3.12 (0.003)	3.04(0.002)	1.01 (1)	**GADD45A**	-2.09 (0.16)	-2.69 (0.22)	1.06 (1)

**BFAR**	3.01 (0.0006)	1.84(0.03)	1.01 (1)	**HRK**	-3.75 (0.01)	-6.11 (0.01)	1.35 (0.30)

**BID**	-1.03 (0.93)	1.16 (0.43)	-1.44 (0.21)	**IGF1R**	-2.29 (0.08)	-3.16 (0.07)	1.14 (1)

**BIK**	1.1 (1)	1.47 (0.34)	-1.61 (0.38)	**LTA**	-7.34 (0.05)	-4.72 (0.01)	-1.88 (0.12)

**BIRC2**	3.77 (0.006)	-7.26 (0.004)	22.63 (0.001)	**LTBR**	-1.38 (0.25)	-1.13 (0.47)	-1.47 (0.27)

**BIRC3**	-3.02 (0.001)	-10.13 (0.004)	2.77 (0.001)	**MCL1**	1.51 (0.33)	1.34 (0.46)	-1.07 (0.51)

**BIRC4**	-11.77 (0.0007)	-15.14 (0.0007)	1.06 (1)	**NAIP**	1.96 (0.13)	1.39 (0.34)	1.16 (1)

**BIRC6**	-1.08 (0.85)	1.04 (0.73)	-1.37 (0.18)	**NOD1**	-1.31 (0.36)	-1.09 (0.94)	-1.44 (0.75)

**BIRC8**	-17.71 (0.0005)	-21.71 (0.0002)	1.01 (1)	**NOL3**	-15.74 (0.001)	-10.34 (0.0003)	-1.84 (0.21)

**BNIP1**	-9.17 (0.0008)	-10.41 (0.001)	-1.06 (1)	**PYCARD**	1.12 (0.48)	1.79 (0.07)	-1.93 (0.05)

**BNIP2**	-8.15 (0.005)	-7.52 (0.007)	-1.31 (0.24)	**RIPK2**	-1.11 (0.21)	-1.69 (0.48)	1.26 (0.08)

**BNIP3**	-8.38 (0.02)	-9.71 (0.03)	-1.04 (1)	**TNF**	2.08 (0.18)	2.3 (0.31)	-1.34 (0.29)

**BNIP3L**	-3.75 (0.02)	-4.41 (0.01)	-1.03 (1)	**TNFRSF10A**	-6.13 (0.013)	-4.03 (0.01)	-1.84 (0.13)

**BRAF**	-1.09 (0.83)	-1.27 (0.15)	-1.04 (1)	**TNFRSF10B**	-5.72 (0.016)	-4.92 (0.02)	-1.4 (0.43)

**CARD6**	-15.74 (0.001)	-19.29 (0.0008)	1.01 (1)	**TNFRSF11B**	-15.74 (0.007)	-19.29 (0.007)	1.01 (1)

**CARD8**	-4.55 (0.01)	-3.76 (0.01)	-1.46 (0.19)	**TNFRSF1A**	2.42 (0.13)	1.65 (0.11)	1.21 (0.76)

**CASP1**	-1.35 (0.45)	1.06 (0.48)	-1.74 (0.01)	**TNFRSF21**	1.1 (0.29)	-1.48 (0.83)	1.35 (0.12)

**CASP10**	-5.12 (0.01)	-6.5 (0.012)	1.05 (1)	**TNFRSF25**	1.07 (1)	3.61 (0.03)	-4.06 (0.03)

**CASP14**	-25.4 (0.03)	-58.89 (0.0004)	1.92 (0.02)	**TNFRSF9**	-15.74 (0.03)	-18.38 (0.01)	-1.04 (1)

**CASP2**	1.05 (1)	1.1 (1)	-1.27 (1)	**TNFSF10**	-1.25 (0.12)	1.45 (0.08)	-2.2 (0.01)

**CASP3**	1.07 (1)	-1.13 (0.68)	-1.01 (1)	**TNFSF8**	-9.04 (0.007)	-10.7 (0.008)	-1.02 (1)

**CASP4**	-1.47 (0.08)	-1.57 (0.09)	-1.13 (0.72)	**TP53**	1.27 (0.20)	1.2 (0.23)	-1.14 (0.70)

**CASP5**	-12.27 (0.002)	-11.71 (0.002)	-1.27 (0.16)	**TP53BP2**	-15.74 (0.02)	-19.29 (0.003)	1.01 (1)

**CASP6**	1.2 (0.34)	1.2 (0.43)	-1.21 (0.99)	**TP73**	-53.32 (0.006)	-48.5 (0.006)	-1.33 (0.70)

Fold changes of 84 AM gene expression, according to the alternative version of 2^-ΔΔCT ^method, for three different comparisons between phenotypic classes: chemoresistant vs control; chemosensitive vs control; chemoresistant vs chemosensitive. When the RQ is ≥1, we reported the same RQ number (positive fold change); when RQ is < 1, we reported -1/RQ: (negative fold change). We considered as up regulated or down regulated those genes with fold change ≥2 and ≤ -2, respectively. Genes with a fold change between 2 and -2 are considered as unchanged. Adjusted Bonferroni p-value is reported between brackets.

**Table 2:** Common differentially expressed genes in chemoresistant and chemosensitive classes in comparison with normal controls

Gene Symbol	Category	Expression	NOTES
BAG3	Anti	Down	Down expression of BAG3 increases apoptosis induced via Bax or Fas by IL-3 deprivation in hematopoietic cells. Furthermore, BAG3 down modulation is recently shown to enhance the apoptotic response to chemotherapy with alkylating agents through regulation of CHK2 and CDC2 proteins in human primary B chronic lymphocytic leukemia cells.
BCL10	Pro	Up	This gene contains a CARD domain, and has been shown to induce apoptosis and to activate NF-kappaB. It is interesting that deregulation of this gene leads to pathogenesis of hematopoietic malignancy. We detected it in HSC from our cohort of patients. Its over expression in CR CD34+ cells could be related to their proneness to death induction.
BCL2L10	Pro	Down	The protein of this gene act as pro-apoptotic regulators that are involved in a wide variety of cellular activities, interacting with other members of BCL-2 protein family including BCL2, BCL2L1/BCL-X(L), and BAX.
BCLAF1	Pro	Up	This gene encodes a transcriptional repressor that interacts with several members of the BCL2 family. Its overexpression induces apoptosis.
BIRC4	Anti	Down	It is a protein which inhibits apoptosis through binding to tumor necrosis factor receptor-associated factors TRAF1 and TRAF2. It also inhibits at least two members of the caspase family of cell-death proteases, caspase-3 and caspase-7.
BIRC8	Anti	Down	BIRC8 is involved in the control of apoptosis by direct inhibition of caspase 9.
BNIP1	Anti	Down	BNIP genes area members of the BCL2/adenovirus E1B 19 kd-interacting protein (BNIP) family. They interact with the E1B 19 kDa protein which is responsible for the protection of virally-induced cell death, as well as, E1B 19 kDa-like sequences of BCL2.
BNIP2	Anti	Down	
BNIP3	Pro/Anti	Down	BNIP3 interacts with the E1B 19 kDa protein which is responsible for the protection of virally-induced cell death, as well as E1B 19 kDa-like sequences of BCL2. The dimeric mitochondrial protein is known to induce apoptosis, even in the presence of BCL2.
CARD6	Anti	Down	This protein is a microtubule-associated protein that has been shown to interact with receptor-interacting protein kinases and positively modulate signal transduction pathways converging on activation of the inducible transcription factor NF-kB.
CASP14	Pro	Down	Caspases encode members of the cysteine-aspartic acid protease (caspase) family. Sequential activation of caspases plays a central role in the execution-phase of cell apoptosis by inducing of either TNF or FAS-receptor.
CASP5	Pro	Down	
CD70	Pro	Down	This cytokine is also reported to play a role in regulating B-cell activation, cytotoxic function of natural killer cells, and immunoglobulin synthesis. Its downexpression could confirm that CD34+ cells from these patients have an expression profile prone to apoptosis and to positively respond to chemotherapy
CFLAR	Anti	Down	c-FLIP inhibits caspase 8 activation and apoptosis mediated by death receptors, such as Fas. Furthermore, overexpression of c-FLIP potently inhibits apoptosis induced by chemotherapy, suggesting that c-FLIP has a role in mediating chemoresistance.
CIDEA	Pro	Down	Cidea that has been shown to activate apoptosis by disrupting a complex consisting of DFF40/CAD.
DAPK1	Pro	Down	This gene commonly results over expressed in hematopoietic malignancies, but it is down regulated in patients after chemotherapy. It could be considered a CR marker.
DFFA	Pro	Up	DFFA is the substrate for caspase-3 and triggers DNA fragmentation during apoptosis.
NOL3	Anti	Down	NOL3, an apoptosis suppressor limited to terminally differentiated cells, is induced in human breast cancer and confers chemo-and radiation-resistance.
TNFRSF11B	Anti	Down	Downexpression of this gene in our model could be considered a CR marker: this gene commonly results over expressed in hematopoietic malignancies, but it is down regulated in patients after chemotherapy
TNFRSF9	Anti	Down	This receptor contributes to the clonal expansion, survival, and development of T cells. It can also induce proliferation in peripheral monocytes, enhance T cell apoptosis induced by TCR/CD3 triggered activation.
TNFSF8	Anti	Down	This gene commonly results over expressed in hematopoietic malignancies, but it is down regulated in patients after chemotherapy. It could be considered a CR marker.
TP53BP2	Pro	Down	This protein is localized to the perinuclear region of the cytoplasm, and regulates apoptosis and cell growth through interactions with other regulatory molecules including members of the p53 family.
TP73	Pro	Down	This gene commonly results over expressed in hematopoietic malignancies, but it is down regulated in patients after chemotherapy. It could be considered a CR marker.

The biological functions, AM category (pro-or anti apoptotic activity), and our comments, based on our findings, are reported for each gene

**Table 3:** Differentially expressed genes in chemoresistant patients respect to chemosensitive patients.

Gene Symbol	Category	Expression	NOTES
APAF1	pro	Down	It is a component of apoptosome. The apoptosome has a role in chemioresistance in pancreatic cancer.
BCL2A1	anti	Down	This gene is able to reduce the release of pro-apoptotic cytochrome c from mitochondria and block caspase activation. This gene is a direct transcription target of NF-kappa B and it acts in response to cytokine TNF and IL-1.
BCL2L1	pro/anti	Down	It acts as anti- or pro-apoptotic regulators. This gene is located on mitochondrial membrane and control VDAC opening, regulating the release of cytochrome C by mitochondria.
BIRC2	anti	Up	This gene is a member of a family of proteins that inhibits apoptosis by binding to tumor necrosis factor receptor-associated factors TRAF1 and TRAF2.
BIRC3	anti	Up	This gene is a member of a family of proteins that inhibits apoptosis by binding to tumor necrosis factor receptor-associated factors TRAF1 and TRAF2. In cells resistant to alkylating agent, it blocks through regulation of CHK2 and CDC2 proteins.
CD40LG	pro	Down	It regulates B cell function by engaging CD40 on the B cell surface.
CIDEB	pro	Down	CIDEB triggers DNA fragmentation and nuclear condensation. Its expression could be associated to resistance to apoptosis.
FAS	pro	Down	FAS/FASL ratio, after receiving chemotherapy, indicates chemosensitivity in several tumoral models. In addition, the decreasing ratio during chemotherapy treatment, despite the initial values, is related to acquired chemoresistance.
FASLG	pro	Down	
TNFRSF25	pro	Down	This receptor has been shown to stimulate NF-kappa B activity and regulate cell apoptosis. The signal transduction of this receptor is mediated by various death domains contained by adaptor proteins.
TNFSF10	pro	Down	This protein preferentially induces apoptosis in transformed and tumoral cells and it is expressed at a significant level in the most of normal tissues. Its down regulation could impair the apoptosis induction.

The biological functions, AM category (pro- or anti apoptotic activity), and our comments, based on our findings, are reported for each gene.

### Specific Features of the AM Network in AML Patients

**Figure 2:**
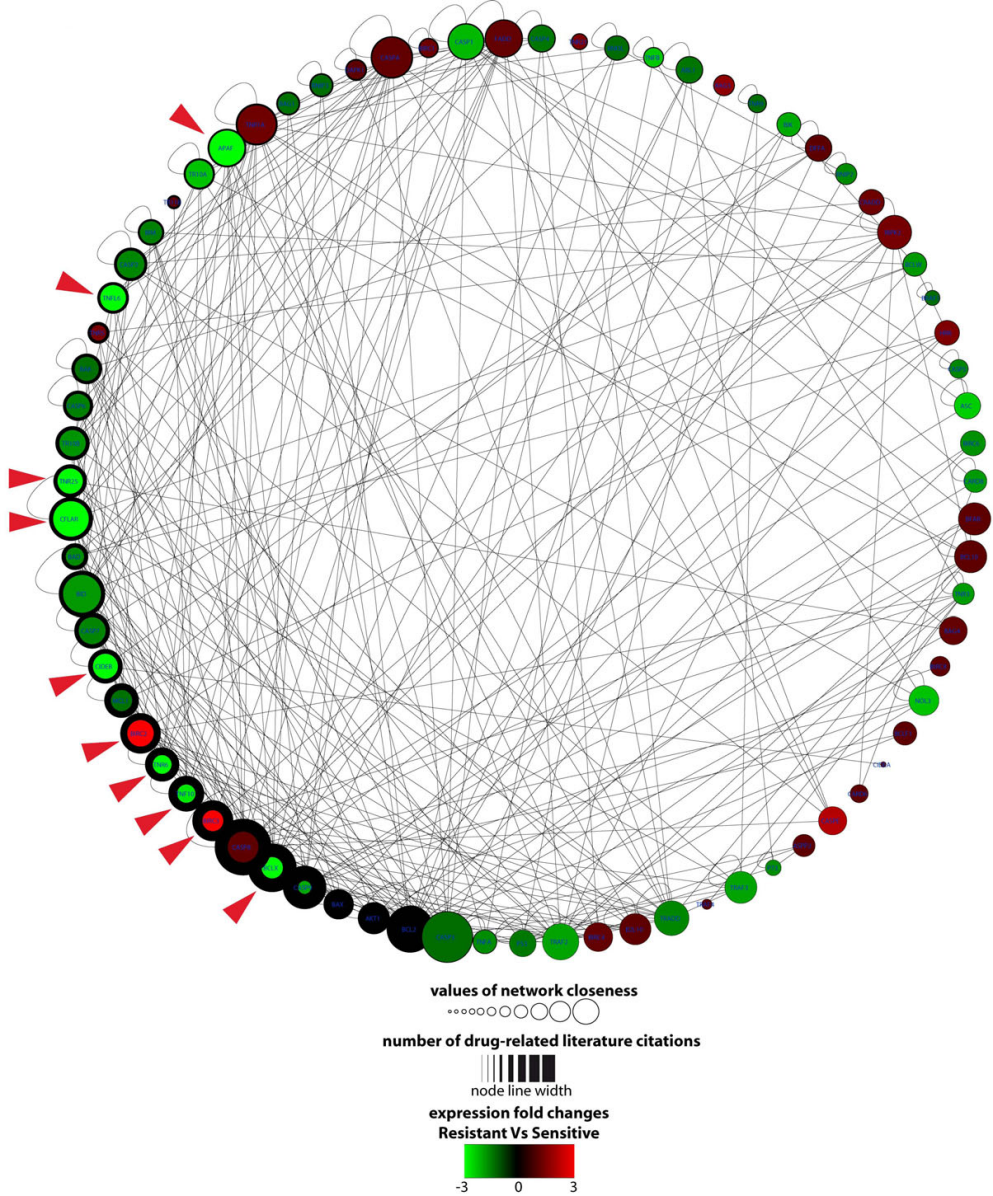
**Correlation among network centrality, expression and involvement in drug response**. yFiles Circular Layout of the AM network that emphasizes the nodes with high closeness and high involvement in drug response. The nodes with high overall drug response (number of literature citations for drugs) are localized on the left half of the circle (attribute circle layout, based on number of literature citations for drugs). The size of the nodes is related to network closeness. The colours indicate the expression fold change of AM genes (chemoresistant vs chemosensitive), according to the bar shown below the network. The node line width indicates the number of literature citations for drugs. As shown by red arrows, all nodes with altered expression are characterized by high closeness and involvement in drug response.

**Figure 3:**
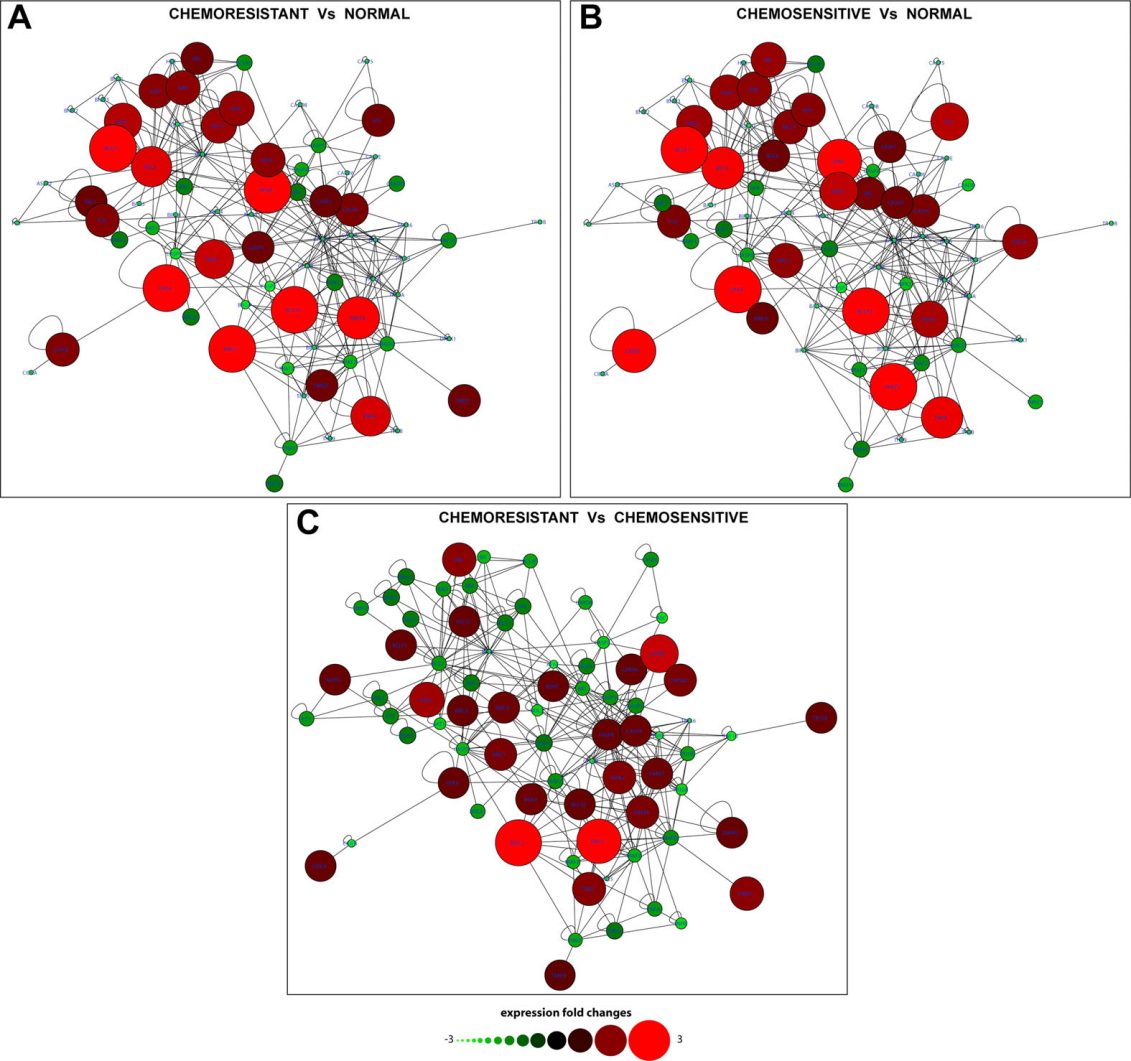
**Different AM network structure based on three different expression profiles**. AM network structure alterations in three different comparisons among chemoresponse classes. A = chemoresistant patients vs controls; B = chemosensitive patients vs controls; C = chemoresistant vs chemosensitive patients. The fold changes are shown by gradients of node colours and sizes, according to the bar shown below the networks. The use of different sizes to show the expression values underlines as different expression profiles could notably alter the structure of a biological network: down regulated nodes have smaller sizes and, accordingly, minor molecular and functional effects on the network; on the other hand, up regulated nodes have larger sizes and a higher potential influence on network functions.

The analysis of network centrality showed that AM genes differentially expressed in *in vitro *resistant CD34+ cells were positively associated to higher network centrality respect to unchanged genes (Wilcoxon Rank Sum test): betweenness (p-value = 0.043), closeness (p-value = 0.019), radiality (p-value = 0.019), stress (p-value = 0.041) (Figure 2). Moreover, these genes were more tightly associated to drug response than unchanged genes (p-value = 0.003) (Figure 2). These network centrality parameters are biologically more important than the simple network degree of a node, because they demonstrate the ability of a protein to functionally connect to and be relevant for several others within a complex signalling network [35]. By comparing chemoresistant and chemosensitive classes, we found that dysregulated genes are frequently critical nodes of the AM network and drug-related genes. Moreover, by integrating the expression values of the three profiles into the network structure, we found that the expression modification of some nodes could lead to important alterations of the network topology (Figure 3). When compared to those from normal donors, the networks of resistant and sensitive classes appeared quite similar; on the contrary, the direct comparison between resistant and sensitive classes identified differences that could unbalance the functional equilibrium of the AM network (e.g., the down regulation of many proapoptotic genes) (Table 3, Figure 3). These altered network structures could explain the different AM behaviour as well as the different *in vitr*o drug sensitivity and the clinical phenotype of patients (i.e., MRD and relapse).

## Discussion

Drug resistance is a major cause of failure in cancer treatment [7,36]. Chemotherapy exerts on tumour cells a strong selective pressure: accordingly, their survival relies on a dynamic mutation process, possibly leading to an ever fitting chemoresistance phenotype [37]. The bone marrow microenvironment is a secluded and potentially safe body niche, in which cancer cells can be protected against chemotoxic agents. Mobilization from this compartment is a complex process, that requires the orchestrated participation of several molecules as chemokines, adhesion molecules, and their downstream targets [10,38]. The molecular events that regulate HSCs' engraftment and mobilization are still incompletely defined. The precise mechanism of mobilization by the Colony Stimulating Factors (CSF), the factor most commonly used to mobilize hematopoietic cells from bone marrow to PB, remains unsatisfactorily characterized [39,40]. CSF binding to its receptor leads to the activation of several downstream signalling cascades affecting cell survival, proliferation, differentiation, migration [41]; moreover, it is well known that CSF suppresses apoptosis in both normal HSCs and cancer cells [42]. In our experiments, we observed that CR patients with high CD34+ mobilization activity showed *in vitro *chemoresistance of their BFU-E and CFU-GM. This was coupled to a poorer prognosis, due to an increased relapse rate respect to normally or highly chemosensitive patients. Intriguingly, chemoresistant patients showed an AM expression profile that strongly suggested the involvement of the AM network in their *in vitro *drug resistance. Thus, a relationship between mobilization from bone marrow, apoptosis induction and chemoresistance seems to exist in these patients. The chemoresistant class is characterized by up regulation of Birc genes (BIRC2 and BIRC3), that inhibit the action of caspases, and down expression of proapoptotic genes as APAF1 (an apoptosome component), FAS, FASL, TNFRSF25, TNFSF10. This molecular phenotype could lead to a failure in activating apoptosis in response to chemotherapy [30-34]. It is well known that GM-CSF inhibits Fas-induced apoptosis and stimulates expression of BIRC family members [43,44]. This protective effect of CSF can also explain the lower complete remission rates after chemotherapy in AML patients, whose cells have a higher responsiveness to hematopoietic viability factors *in vitro *[45]. Our data demonstrate a down regulation of proapoptotic genes CD40LG, CIDEB, TNFSF10 in chemoresistant AML patients, previously not reported in association with drug resistance. Furthermore, differently from previous reports that *in vivo *resistant AML cells express high levels of BCL2A1 and BCL2L1 [30,34], we found that both genes are down regulated in chemoresistant AML patients in comparison to those from the chemosensitive class. We suggest that this discrepancy is due to the specific features of the different cell types analyzed: we analyzed *in vitro *selected chemoresistant, possibly non-leukemic CD34+ cells from CR AML patients, whereas Eisele et al. and Valdez et al. studied myeloid blasts from AML patients [46] or mononuclear cells from AML patients [31], respectively. Why are some CR AML patients *good responders *to CSF and others are not? The continued pressure of chemotherapy may induce or select genomic mutations, able to alter the cytokine cross-talking network between cell migration and apoptosis: this would result in an acquired drug resistance phenotype [8]. We may otherwise assume that within the HSC population of some CR patients, few leukemic cells survive chemotherapy and persist under the CR threshold to be eventually selected by the mobilizing treatment [47,48]. The presence of a substantial number of MRD cells could influence the microenvironment and enhance their protective effect from chemotherapy damage. This would explain not only the high relapse rate, but also the high mobilization activity through a reduced myelotoxicity induced by chemotherapy. Based on our results, we suggest that the presence of MRD can lead to a modulation of BM microenvironment that could cause dysregulation of some components of the AM network in HSC. Interestingly, by comparing both the resistant and the sensitive class with control samples, we noticed that 23 AM genes showed an expression profile common to both resistant and sensitive classes and different respect to controls: many of these genes are involved in an increased response to chemotherapy (e.g., the down regulation of BAG3, BIRC4, BIRC8, BNIP family, CFLAR) [49,50]. Furthermore the downregulation of DAPK1, TNFRSF11B, TNFSF8, TP73 could be considered as a CR marker: these genes are commonly over expressed in hematopoietic malignancies, but are down regulated in patients after chemotherapy [24-26]. The detection of proapoptotic BCL10 in CR CD34+ cells, but not in normal donors (Tables 1 and 3; Figure 1), could be related to their proneness to death induction. Dysregulation of this gene is known to be involved in some types of haematological malignancies [51]. These observations suggest that non-leukemic CD34+ cells from CR AML patients have an expression profile apparently prone to apoptosis and to a positive response to chemotherapy. This molecular feature satisfactorily explains their positive response to *in vivo *chemotherapy induction, leading these patients to the first apparent CR. Plausibly, the molecular differences observed between chemoresistant and chemosensitive (acquired *de novo *mutations or residual presence of leukemic cells) could be responsible for the *de novo *acquired resistant phenotype, and accordingly for the relapse. Unsurprisingly, these differentially expressed genes are characterized by high centrality within the AM network. Furthermore, many published data link these genes to drug response, suggesting that they perform a critical role within AM signalling activated by pharmacological treatments. Taken together these data strongly suggest that the topology of AM network is strictly related to its proper biological functioning. Malfunctioning of these central nodes affects the stability of the network and profoundly modify the physiological cell behaviour. In agreement with other published data, our results suggest that tumour-related defects in AM hubs are preferentially selected [16,52]. The functional impairment of a few nodes, which control directly or indirectly the activities of many others in the context of the co-occurrence of multiple genetic defects, could represent a selective advantage during neoplastic transformation and in response to pharmacological treatment. The differential expression of topologically important AM nodes in CR AML patients could seriously impair the physiological equilibrium of AM. CD34+ cells from both classes of CR AML patients showed AM network features that make these cells prone to apoptosis: accordingly, it is not surprising that these patients were in apparent CR after the first cycle of chemotherapy induction and consolidation (Figure 3). On the other hand, the AM network of the resistant class showed some critical features respect to that of the sensitive class: by impairing proapoptotic components and activating prosurvival nodes, these differences could reduce the ability of these cells to appropriately respond to death stimuli (Figure 3). Some of these AM genes are known to be dysregulated in leukaemia: up regulation of BIRC3 and down regulation of APAF1, CIDEB, FAS, TNFRSF25, TNFSF10 were previously identified by our group as specific alterations of AM genes in leukemic cells [16].

## Conclusions

The ability of some cancer cell types to elude pharmacological apoptosis induction is apparently based on molecular mechanisms, similar to those involved in escaping physiological cell death. In this *scenario*, some AM genes represent critical nodes for the proper response to chemotherapy. Their dysregulation in HSCs from CR AML patients could be related to their high mobilizing ability, *in vitro *chemoresistance and high relapse rates. This molecular phenotype could result from *de novo *mutations, selected by the treatment, or be due to residual leukemic cells, positively selected by the cytokines used for mobilization. The AM expression profile of CD34+ cells seems to discriminate CR AML patients from normal controls, as well as *in vitro *chemoresistant CR AML patients from those who are sensitive. These differences affect some critical nodes of the AM network and could represent one of the causes of the differential *in vitro *resistance of these cells. Accordingly, AM profiling before chemotherapy and transplantation could allow the identification of patients with a genotype highly predisposing to relapse, in order to treat them by different, specifically designed, therapies. Specifically, low expression levels of APAF1, CD40LG, CIDEB, FAS, FASL, TNFRSF25, TNFSF10 and up regulation of BIRC2 and BIRC3 could pinpoint patients who are prone to relapse. Otherwise, the characterization of the genotype of the cells from PB could demonstrate the characteristic molecular signature of leukemic cells and accordingly guide the design of the therapeutic strategy.

## Abbreviations

AM: Apoptotic Machinery; AML: Acute Myeloid Leukaemia; BFU-E: Burst Forming Units Erythroid; BM: Bone Marrow; CFU-E: Colony-Forming-Units Erythroid; CFU-GM: Colony Forming Unit Granulocyte-Macrophage; CR: Complete Remission; CSF: Colony-Stimulating Factor; G-CSF: Granulocyte Colony-Stimulating Factor; GM-CSF: Granulocyte-Macrophage Colony-Stimulating Factor; HSC: Hematopoietic Stem Cells; MRD: Minimal Residual Disease; PB: Peripheral Blood; VLA: Very Late Antigen.

## Competing interests

The authors declare that they have no competing interests.

## Authors' contributions

MP conceived and coordinated the project with the critical collaboration of MR and GM. MP, MR, GM, CDP designed experiments, the other Researchers performed them. All Authors contributed to the critical revision of the data. MP and MR wrote the paper, that was revised and approved by all Authors (MR, GA, RA, DB, MRG, LRD, AM, LS, LS, CC, MGC, CDP, GM, MP).

## Pre-publication history

The pre-publication history for this paper can be accessed here:

http://www.biomedcentral.com/1471-2407/10/377/prepub

## Supplementary Material

Additional file 1 **Clinical characteristics of the patients**. Click here for file
