# Supplementary material for: Expression profile and specific network features of the apoptotic machinery explain relapse of acute myeloid leukemia after chemotherapy
Source: BMC Cancer. 2010 Jul 19;10:377. doi: 10.1186/1471-2407-10-377 (PMC2914706; doi:10.1186/1471-2407-10-377)
Supplement: Additional file 1 — Clinical characteristics of the patients. [file 1471-2407-10-377-S1.PDF]

	Chemoresistant group			***P-value Resistent vs sensitive***	Chemosensitive group			***P-value***		Normal control (*)	
								Resistent vs normal control	***Sensitive vs normal control***		
PATIENT	C P	S N	C C		P V	A B	P R			DV	CC
Age	53	26	52		46	57	44			38	42
Age median	43,6			0,8273	49			0,5637	0,0833	40	
Gender	M	F	F		F	M	M			M	F
FAB	BICLONAL	M5	M1		M4	M1	M4			/	/
WBC at diagnosis	2,8 x 10^9/L	6,8 x 10^9/L	6,3 x x 10^9/L		42,8 x 10^9/L	26 x 10^9/L	16,7 x 10^9/L			/	/
WBC at diagnosis median	5,3 x 10^9/L			0,0495	28,5 x 10^9/L					/	
CD34+ Peak in P.B.	315 mmc	1288 mmc	980 mmc		5,9 mmc	36 mmc	199 mmc			/	/
CD34+ Peak in P.B. median	861 mmc			0,0495	80,3 mmc					/	
Cytogenetics groups	Poor	Poor	Intermediate		Intermediate	Poor	Intermediate			/	/
FLT3 status	/	Wild type	Mutated		/	/	Mutated			/	/
NPM status	/	/	Wild type		Wild type	Wild type	Mutated			/	/
Residual CFU-GM growth at 50 mcg/ml of Maphosphamide	42 %	41 %	67 %		0	13 %	0			35	37
Residual CFU-GM growth at 50 mcg/ml of Maphosphamide median	50 %			0,0495	4,3 %			0,0833	0,0833	36 %	
Residual BFU-E growth at 50 mcg/ml of Maphosphamide	96 %	66 %	/		5 %	8 %	/			23	19

Residual BFU-E growth at 50 mcg/ml of Maphosphamide median	81 %			0,1213	6,5 %			0,1213	0,1213	21 %	
Platelets (in CR after consolidation)	166×10^9/L	277×10^9/L	272×10^9/L		/	192×10^9/L	128×10^9/L			/	/
Platelets (in CR after consolidation) median	$238,3 \times10^9$/L			0,2482	160×10^9/L					/	
WBC (in CR after consolidation):	$5,2 \times10^9$/L	$7,1\times10^9$/L	$8,0 \times10^9$/L		/	$7,9\times10^9$/L	$9,5 \times10^9$/L			/	/
WBC (in CR after consolidation): median	$6,8 \times10^9$/L			0,2482	$8,7 \times10^9$/L					/	
Minimal residual disease (evaluated by leukemic associated immunophenotiping)	2%	1%	1%		0,4%	5%	4,9%			/	/
Minimal residual disease (evaluated by leukemic associated immunophenotiping) median	1,3%			0,5127	3,4%					/	

Personal and clinical data of patients and control donors analyzed in AM profiling. Single values, median and p-values are reported. FAB: French-American-British classification; FLT3: fms-related tyrosine kinase 3; NPM: nucleophosmin; WBC: White Blood Cell Count.
(*) In a dataset of 12 normal donors, the residual growth of the CFU-GM treated with 50 mcg/ml of Mafosfamide is comprised between 20% and 40%. The patients were classified in chemosensitive if they showed a residual growth of the CFU-GM lower than 20%; while were considered chemoresistant if this value was higher than 40%.
